# Parasitic Contamination of Fresh Vegetables Marketed and Cultivated in Small‐Scale Farms in Hosanna Town and Its Surrounding Areas, Hadiya Zone, Ethiopia

**DOI:** 10.1155/japr/6786335

**Published:** 2026-07-31

**Authors:** Gebremedhn Yohannes, Amha Gebremariam, Yoseph Baraki

**Affiliations:** ^1^ Department of Biology, College of Natural and Computational Science, Wachemo University, Hosanna, Ethiopia, wcu.edu.et

**Keywords:** parasitic contamination, postharvest, preharvest, vegetables

## Abstract

Fresh vegetables are important components of a healthy diet but may serve as vehicles for transmission of intestinal parasites when contaminated during production, harvesting, transport, and marketing. This study assessed the prevalence and distribution of parasitic contamination in fresh vegetables cultivated on small‐scale farms and marketed through wholesale and retail outlets in Hosanna town and surrounding areas. A cross‐sectional study was conducted from December 2024 to June 2025 on 492 vegetable samples, including Ethiopian kale, Swiss chard, carrot, tomato, cabbage, and beetroot, collected from small‐scale farms (*n* = 96), wholesale markets (*n* = 216), and retail markets (*n* = 180). Samples were washed with physiological saline and microscopically inspected for intestinal parasite infectious stages. The study revealed that parasitic contamination increased significantly along the farm (preharvest)‐to‐market (postharvest) chain (*p* < 0.05), with 35.42% of preharvest farm samples, 46.30% of wholesale, and 72.78% of retail market (postharvest) samples testing positive. Among farm (preharvest) samples, Ethiopian kale (54.16%) and beetroot (41.66%) had the highest contamination rates, predominantly with *Strongyloides stercoralis* (33.33%) and *Entamoeba histolytica*/*dispar* (16.67%). In wholesale markets (postharvest), carrot showed the highest contamination (69.44%), whereas *E. histolytica/dispar* was the most frequently detected parasite (30.56%). Retail markets (postharvest) exhibited the greatest contamination burden, particularly tomatoes and cabbages from Arada and Gofer Meda markets, where *E. histolytica/dispar* reached 50.00%. Leafy and root vegetables, especially Ethiopian kale and carrots, were the most contaminated with parasites. Fresh vegetables grown and sold in the study area were commonly contaminated with intestinal parasites, with retail vegetables showing the highest contamination rates. Enhanced hygiene practices during production, handling, and marketing, alongside targeted public health interventions, are crucial to reducing foodborne parasitic infections in the community.

## 1. Introduction

Fresh vegetables are an essential component of healthy diets because they provide vitamins, minerals, dietary fiber, antioxidants, and other bioactive compounds that reduce the risk of chronic diseases and support overall human health [1, 2]. However, the ingestion of raw or inadequately washed vegetables is a major risk factor for foodborne diseases due to the presence of parasites (protozoa and helminths), thereby posing substantial public health risks worldwide [2–4]. Globally, foodborne parasitic diseases remain a major concern, particularly in low‐ and middle‐income countries where sanitation systems, wastewater treatment, and food safety infrastructure are limited [4, 5]. Recent evidence indicates that contamination of fresh vegetables with intestinal parasites is widespread across Asia, Africa, the Middle East, and Latin America, with prevalence ranging from approximately 20% to over 80% depending on region, vegetable type, environmental sanitation, and diagnostic methods [2, 6–8].

Systematic reviews and regional studies have shown marked geographic differences in parasitic contamination of vegetables. Studies from Africa often report higher contamination burdens than those from many developed regions, largely due to unsafe irrigation practices, use of untreated animal manure, poor sanitation, and inadequate postharvest hygiene [4, 9]. For example, contamination rates of 75.2% in Nigeria, 67.5% in wastewater‐irrigated vegetables in Addis Ababa, and over 60% in several Ethiopian urban markets have been reported [10–12]. In contrast, lower prevalence has been documented in some parts of Türkiye (26.2%) and Morocco, where relatively improved sanitary infrastructure may reduce contamination [13, 8]. Commonly identified parasites across global studies include *Entamoeba histolytica/dispar*, *Giardia lamblia*, *Ascaris lumbricoides*, *Strongyloides stercoralis*, hookworms, *Taenia* spp., *Trichuris trichiura*, and *Cryptosporidium* spp. [4, 8, 10]. These parasites are of considerable medical importance because they may cause gastrointestinal disease, malnutrition, anemia, and broader socioeconomic burdens.

Vegetable contamination can occur throughout the farm‐to‐fork continuum. Preharvest contamination is often associated with irrigation using untreated wastewater, contaminated river water, use of raw animal manure, soil contamination, and inadequate field sanitation [5, 14]. Postharvest contamination may intensify through handling by infected vendors, poor washing practices, contaminated transport containers, prolonged market display, exposure to dust and flies, and direct placement on contaminated surfaces [3, 15]. Leafy vegetables such as lettuce, cabbage, spinach, and Ethiopian kale are frequently more contaminated than smooth‐surfaced vegetables because their uneven surfaces facilitate adherence of parasite stages [4, 15]. Root vegetables may also carry substantial parasite loads because of direct soil contact [8].

In Ethiopia, where small‐scale farming supplies much of the domestic vegetable market, parasitic contamination of vegetables represents a persistent and significant public health challenge. A recent nationwide systematic review and meta‐analysis estimated that approximately 43.4%–44% of raw vegetables and fruits in Ethiopia are contaminated with medically important parasites, with *Entamoeba* spp., *Ascaris* spp., *Giardia* spp., and *Strongyloides* spp. among the most prevalent contaminants [4, 14]. Studies conducted in Jimma, Arba Minch, Addis Ababa, Debre Berhan, Wolkite, and Butajira have consistently documented high contamination rates, though prevalence varies by agroecology, market systems, and hygiene practices [3, 11, 12, 15]. Despite this growing body of evidence, many studies have focused primarily on market‐level contamination, with limited comparative assessment of contamination dynamics between preharvest small‐scale farms, wholesale markets, and retail outlets.

Understanding contamination differences across production and marketing stages is particularly important because wholesale markets may reflect contamination originating mainly from farms and transportation systems, whereas retail markets may demonstrate additional contamination from repeated handling, environmental exposure, and poor sanitation [3, 15]. Comparing these stages provides critical insight into where interventions may be most effective, whether at production, transport, or final sale points.

Hosanna town and its surrounding Hadiya Zone are characterized by intensive small‐scale vegetable farming and active informal vegetable trade, yet there is limited evidence regarding parasitic contamination across this full farm‐to‐market pathway. Local agricultural practices such as river–water irrigation, animal manure use, and informal retail marketing may increase contamination risks, but these factors have not been sufficiently investigated in this setting. Therefore, this study is aimed at assessing and comparing the prevalence, diversity, and distribution of intestinal parasites contaminating fresh vegetables at preharvest (small‐scale farms) and postharvest (wholesale and retail markets) levels in Hosanna town and surrounding areas.

## 2. Materials and Methods

### 2.1. Description of the Study Area

The study was conducted at Hosanna town and the surrounding small‐scale farms, Hadiya Zone, Ethiopia. Specific wholesale and retail markets (Gofer Meda and Naremo) in Hosanna and selected small‐scale farms from Doyogena and Wonjela Meda. Hosanna town, which is the administrative and commercial center of the Hadiya Zone, is located approximately 232 km south of Addis Ababa, the capital city of Ethiopia. Geographically, the town lies at 7°30 ^′^ N latitude and 37°48 ^′^ E longitude. The area is situated at an elevation ranging from 1900 to 2700 m.a.s.l. and covers an estimated area of 40.5 km^2^. Hosanna has a moderate subtropical highland climate, with an annual rainfall of 1001–1200 mm being the case. The mean annual temperature ranges from 15.1°C to 20°C, creating favorable conditions for small‐scale vegetable cultivation and other agricultural activities.

### 2.2. Study Design and Sampling Strategy

An exploratory cross‐sectional design was used to assess the presence and prevalence of parasitic contamination on selected fresh vegetables sold at preharvest (farm) and postharvest (wholesale and retail market) stages. Purposive site selection was used to identify accessible farms and major markets with high vegetable flow. Within these selected sites, vegetable samples were collected based on availability during the study period. Samples of preharvest vegetables were collected from small‐scale farms and postharvest from wholesale and retail markets of Hosanna town from December 2024 to June 2025.

### 2.3. Target Population and Sample Selection

#### 2.3.1. Target Population

Fresh vegetables are commonly consumed raw or after minimal processing by the local population and are available in markets and farms. Typical vegetables include Swiss chard (*Beta vulgaris* L.), Ethiopian kale (*Brassica carinata*), carrot (*Daucus carota*), tomato (*Lycopersicon esculentum*), cabbage (*Brassica oleracea*), and beetroot (*Beta vulgaris* var. *esculenta*).

### 2.4. Inclusion and Exclusion Criteria

#### 2.4.1. Inclusion

Fresh vegetable samples were sold in selected wholesale and retail markets, and freshly harvested vegetables from small‐scale farms during the study period. Samples must be whole and not processed.

#### 2.4.2. Exclusion

The exclusion criteria include prewashed, packaged, or visibly spoiled/rotten vegetables.

### 2.5. Sample Size and Sample Collection Procedure

A total of 492 vegetable samples were examined, including 96 preharvest samples from farms and 396 postharvest samples from wholesale and retail markets. Sample numbers were determined pragmatically based on site accessibility, vegetable availability, and logistical feasibility across the study period. Selection of samples from markets and small farmlands was based on the availability of such vegetables. The market samples were composed of 216 from Hosanna′s main market and 180 from retail markets, that is, Arada (*n* = 60), Gofer Meda (*n* = 60), and hospital (*n* = 60) markets. Commonly consumed vegetables, including Swiss chard (*Beta vulgaris* L.), Ethiopian kale (*Brassica carinata*), carrot (*Daucus carota* L.), tomato (*Lycopersicon esculentum*), cabbage (*Brassica oleracea*), and beetroot (*Beta vulgaris*), were collected randomly from selected Hosanna town markets and small farmlands. Vegetable samples were collected from available vendors and farms using standardized handling procedures. Each sample consisted of approximately 500 g of whole, visibly fresh, unprocessed vegetable material. Vegetable samples were collected in triplicate from each farm and market seller using separate sterile plastic bags and were transported on the same day to the laboratory of the Department of Biology, Wachemo University.

### 2.6. Survey for Demographic Information and Agronomic Practices

Structured questionnaires were administered to participating farmers and vendors to collect information on sociodemographic characteristics, irrigation sources, fertilizer use, transportation practices, hygiene behaviors, vegetable display methods, and awareness of contamination risks.

### 2.7. Detection of Helminth Eggs, Larvae, and Protozoan Cysts

Parasitological examination was performed using saline washing, sedimentation, centrifugation, and Lugol′s iodine wet mount microscopy. Parasites from vegetables were detected by taking 200 g of each fresh vegetable sample. Samples were chopped into small pieces and washed in 500 mL physiological saline solution (0.85% NaCl) for 15 min separately. After removing vegetable fragments from the washing saline using clean forceps, the washing was left for 24 h for sedimentation to take place. After 24 h of sedimentation, the top layer of the washing saline was carefully discarded without shaking, and 5 mL of the sediment was centrifuged at 2000 rotations/min. The final sediment was examined microscopically after Lugol′s iodine staining for the presence of helminth eggs, larvae, and protozoan cysts [13, 16].

### 2.8. Statistical Analysis

Data were analyzed using SPSS Version 20. Descriptive statistics were used to summarize contamination frequencies and prevalence patterns. Chi‐square tests were applied to compare contamination rates across vegetable types and sampling sources. Independent samples *t*‐tests were used for selected comparisons between preharvest and postharvest groups. The differences were considered significant at *p* < 0.05.

## 3. Result

### 3.1. Sociodemographic Characteristics and Vegetable‐Handling Practices

A total of 161 respondents (51 farmers and 110 vendors) participated in the study (Table 1). Farmers were predominantly male, whereas vendors were mainly female, reflecting gender‐based roles across the production and marketing chain. Most respondents were economically active adults aged 31–45 years, and primary education was the most common educational level. Nearly half had 5–10 years of experience in vegetable farming or selling.

**Table 1:** Sociodemographic characteristics and key handling practices of farmers and vendors in Hosanna town and surrounding areas.

Variable	Category	Farmers, *n* (%)	Vendors, *n* (%)	Total, *n* (%)
Sex	Male	38 (74.5)	32 (29.1)	70 (43.48)
	Female	13 (25.5)	78 (70.9)	91 (56.52)
Age	18–30 years	6 (11.8)	31 (28.2)	37 (22.98)
	31–45 years	22 (43.1)	46 (41.8)	68 (42.24)
	46–60 years	17 (33.3)	24 (21.8)	41 (25.47)
	> 60 years	6 (11.8)	9 (8.2)	15 (9.32)
Educational status	Illiterate	10 (19.6)	20 (18.2)	30 (18.63)
	Primary education	24 (47.1)	41 (37.3)	65 (40.37)
	Secondary education	12 (23.5)	33 (30.0)	45 (27.95)
	College and above	5 (9.8)	16 (14.5)	21 (13.04)
Occupation	Farmer	51 (100)	—	51 (31.68)
	Vegetable vendor	—	110 (100)	110 (68.32)
Experience	< 5 years	10 (19.6)	36 (32.7)	46 (28.57)
	5–10 years	25 (49.0)	53 (48.2)	78 (48.45)
	> 10 years	16 (31.4)	21 (19.1)	37 (22.98)
Fertilizer type	Synthesized fertilizer	16 (31.4)	—	—
	Animal manure	27 (52.9)	—	—
	Compost	8 (15.7)	—	—
	Municipal sewage	0 (0)	—	—
Use of protective materials	Yes	0 (0)	—	—
	No	51 (100)	—	—
Source of vegetables	Small farm	51 (100)	—	—
	Garden	0 (0)	—	—
Irrigation water source	River	30 (58.82)	—	—
	Well	16 (31.37)	—	—
	Tap water	5 (9.8)	—	—
Awareness of contamination risk	Aware	15 (29.41)	—	—
	Not aware	36 (70.59)	—	—
Source of vegetables	Market	—	110 (100)	—
	Farm	—	0 (0)	—
Market sanitation	Poor	—	60 (54.55)	—
	Fair	—	33 (30.0)	—
	Good	—	17 (15.45)	—
Transportation system	Domestic ass cart	—	56 (50.9)	—
	Handcart	—	11 (10.0)	—
	Cooly	—	29 (26.5)	—
	Vehicles	—	14 (12.7)	—
Washing before display	Washed	—	39 (35.45)	—
	Unwashed	—	73 (66.36)	—
Display method	On ground	—	72 (65.45)	—
	On table/shelf	—	38 (34.55)	—
Awareness of contamination risk	Aware	—	35 (31.18)	—
	Not aware	—	75 (68.48)	—
Use of protective materials	Yes	—	0 (0)	—
	No	—	110 (100)	—
Storage duration	< 2 days	—	0 (0)	—
	2–4 days	—	47 (42.7)	—
	5–7 days	—	63 (57.3)	—
	> 7 days	—	0 (0)	—

Production and marketing practices revealed multiple contamination risks. Among farmers, over half used animal manure as fertilizer, most relied on river water for irrigation, and none reported using protective materials during harvesting or handling. Awareness of contamination risks was low among farmers (29.4%). Similarly, among vendors, poor sanitation conditions, transport by donkey cart or manual carriers, display of vegetables on the ground, limited washing before sale, prolonged storage, and complete absence of protective equipment were common. Vendor awareness of contamination risks was also limited (31.2%). Overall, these results suggest that hygiene shortcomings at both the preharvest and postharvest stages may contribute to the risk of parasitic contamination.

### 3.2. Overall Prevalence of Parasitic Contamination by Source

A total of 492 vegetable samples were examined from preharvest farmlands, wholesale markets, and retail markets to assess parasitic contamination (Table 2). Parasitic contamination increased progressively along the farm‐to‐market chain. Overall, 277 (56.3%) of the samples were contaminated with at least one parasitic species. Of the 96 preharvest vegetable samples collected from small‐scale farms, 35.42% were contaminated with at least one intestinal parasite. Postharvest contamination was substantially higher, affecting 46.30% of wholesale market samples and 79.44% of retail market samples. Retail markets showed the highest contamination burden, suggesting that repeated handling, poor market hygiene, and prolonged exposure substantially increased risk. Across all sources, *E. histolytica/dispar* and *S. stercoralis* were the dominant contaminants.

**Table 2:** Overall prevalence of parasitic contamination of vegetables by source.

Source of vegetables	Sampling location	No. examined	No. positive	Contamination rate (%)	Most contaminated vegetable(s)	Predominant parasites detected
Preharvest (farmlands)	Doyogena and Wonjela Meda	96	34	35.42%	Ethiopian kale (54.16%)	*Strongyloides stercoralis*
Wholesale market (postharvest)	Hosanna town wholesale market	216	100	46.30%	Carrot (69.44%)	*E. histolytica/dispar*, *S. stercoralis*
Retail markets (postharvest)	Arada, Gofer Meda, hospital markets	180	143	79.44%	Tomato and cabbage (50%)	*E. histolytica/dispar*
Total	—	492	277	56.3%	—	*—*

### 3.3. Prevalence of Intestinal Parasites in Raw Vegetables From Preharvest (Farmlands) and Postharvest (Wholesale and Retail Markets) Samples

The findings presented in Table 3 demonstrated considerable variation in parasite distribution among different vegetable types and sampling sources. Among all parasites detected, *E. histolytica/dispar* showed the highest prevalence in tomato samples obtained from retail markets, accounting for 47.2% of examined samples. Similarly, Ethiopian kale and cabbage from retail markets also showed high contamination rates with *E. histolytica/dispar* (30.6% and 33.3%, respectively).

**Table 3:** Distribution of parasites in samples collected from preharvest farmlands (*n* = 24), postharvest wholesale markets (*n* = 36), and retail markets (*n* = 36).

Raw vegetables	Source	*Entamoeba histolytica/dispar*	*Strongyloides stercoralis*	*Taenia* spp.	Hookworm	*Ascaris lumbricoides*	*χ* ^2^(vegetables vs. parasites)	*p* value
Ethiopian kale	Farmlands	7 (29.2%)	3 (12.5%)	2 (8.3%)	0 (0%)	1 (4.2%)	12.26	0.481
	Wholesale markets	11 (30.6%)	4 (11.1%)	0 (0%)	5 (13.9%)	0 (0%)	166.26	0.001
	Retail markets	11 (30.6%)	5 (13.9%)	6 (16.7%)	7 (19.4%)	5 (13.9%)	166.26	0.001
Tomato	Farmlands	NE	NE	NE	NE	NE	—	—
	Wholesale markets	4 (11.1%)	2 (5.6%)	0 (0%)	0 (0%)	0 (0%)	205.56	0.03
	Retail markets	17 (47.2%)	5 (13.9%)	1 (2.8%)	5 (13.9%)	2 (5.6%)	205.56	0.03
Cabbage	Farmlands	1 (4.2%)	1 (4.2%)	1 (4.2%)	0 (0%)	0 (0%)	7.80	0.072
	Wholesale markets	4 (11.1%)	0 (0%)	0 (0%)	2 (5.6%)	0 (0%)	377.81	0.0001
	Retail markets	12 (33.3%)	9 (25.0%)	6 (16.7%)	9 (25.0%)	3 (8.3%)	377.81	0.0001
Carrot	Farmlands	0 (0%)	4 (16.7%)	0 (0%)	2 (8.3%)	0 (0%)	0.46	0.050
	Wholesale markets	6 (16.7%)	15 (41.7%)	0 (0%)	4 (11.1%)	0 (0%)	255.15	0.0005
	Retail markets	3 (8.3%)	10 (27.8%)	0 (0%)	6 (16.7%)	3 (8.3%)	255.15	0.0005
Beetroot	Farmlands	0 (0%)	8 (33.3%)	2 (8.3%)	0 (0%)	0 (0%)	3.83	0.039
	Wholesale markets	0 (0%)	9 (25.0%)	0 (0%)	8 (22.2%)	0 (0%)	327.26	0.049
	Retail markets	6 (16.7%)	9 (25.0%)	1 (2.8%)	2 (5.6%)	2 (5.6%)	327.26	0.049
Swiss chard	Farmlands	NE	NE	NE	NE	NE	—	—
	Wholesale markets	8 (22.2%)	0 (0%)	0 (0%)	2 (5.6%)	13 (36.1%)	69.16	0.001
	Retail markets	NE	NE	NE	NE	NE	—	—

Abbreviation: NE, not examined.

*S. stercoralis* was the dominant parasite in carrot and beetroot samples. The highest prevalence was recorded in carrot samples from wholesale markets (41.7%), followed by beetroot samples from farmlands (33.3%) and retail markets (25.0%). Hookworm contamination was highest in beetroot samples from wholesale markets (22.2%) and Ethiopian kale from retail markets (19.4%). The presence of hookworm in vegetables indicates possible contamination from fecally polluted soil or irrigation water. Similarly, *Taenia* spp. contamination was highest in Ethiopian kale and cabbage from retail markets (16.7% each), whereas the least occurrence was observed in several wholesale and farmland samples, where no contamination was detected.

*A. lumbricoides* was most prevalent in Swiss chard samples from wholesale markets (36.1%), representing the highest prevalence of this parasite among all vegetable types examined. Conversely, *A. lumbricoides* was absent in most farmland and wholesale samples of carrot, cabbage, beetroot, and tomato, indicating comparatively lower contamination levels. The least parasite contamination overall was observed in cabbage and carrot farmland samples. For example, cabbage farmland samples showed only 4.2% contamination for *E. histolytica/dispar*, *S. stercoralis*, and *Taenia* spp., whereas carrot farmland samples had no detectable contamination with *E. histolytica/dispar*, *Taenia* spp., or *A. lumbricoides*. In addition, several vegetables such as tomato and Swiss chard were not examined at the farmland and retail levels, limiting comparisons across all sources.

### 3.4. Comparative Prevalence of Intestinal Parasites in Raw Vegetables From Wholesale and Retail Markets

The comparative analysis of intestinal parasite contamination in raw vegetables collected from wholesale and retail markets in Hosanna revealed that retail market vegetables generally had higher levels of contamination, 131 (72.78%), than wholesale market vegetables, 100 (46.30%) (Table 4). Among the parasites identified, *E. histolytica/dispar* showed notably higher prevalence in retail market vegetables compared with wholesale markets. The highest contamination was observed in retail tomatoes (47.2%), followed by retail cabbage (33.3%). In contrast, wholesale samples of tomato and cabbage each showed only 11.1% contamination, whereas beetroot samples from wholesale markets had no detectable contamination with *E. histolytica/dispar*. The chi‐square analysis demonstrated statistically significant associations between vegetable types and parasite occurrence for all examined parasites, including *E. histolytica/dispar* (*χ* ^2^ = 166.26, *p* = 0.001), *S. stercoralis* (*χ* ^2^ = 205.56, *p* = 0.03), *Taenia* spp. (*χ* ^2^ = 377.81, *p* = 0.0001), hookworm (*χ* ^2^ = 255.15, *p* = 0.00005), and *A. lumbricoides* (*χ* ^2^ = 327.26, *p* = 0.049). These results confirm significant differences in parasite distribution between vegetable types and market sources.

**Table 4:** Comparative prevalence of intestinal parasites in raw vegetables from wholesale and retail markets in Hosanna, Ethiopia.

Parasites	Ethiopian kale wholesale (*N* = 36)	Ethiopian kale retail (*N* = 36)	Tomato wholesale (*N* = 36)	Tomato retail (*N* = 36)	Cabbage wholesale (*N* = 36)	Cabbage retail (*N* = 36)	Carrot wholesale (*N* = 36)	Carrot retail (*N* = 36)	Beetroot wholesale (*N* = 36)	Beetroot retail (*N* = 36)	*χ* ^2^	*p* value
*Entamoeba histolytica/dispar*	11 (30.6%)	11 (30.6%)	4 (11.1%)	17 (47.2%)	4 (11.1%)	12 (33.3%)	6 (16.7%)	3 (8.3%)	0 (0%)	6 (16.7%)	166.26	0.001
*Strongyloides stercoralis*	4 (11.1%)	5 (13.9%)	2 (5.56%)	5 (13.89%)	0 (0%)	9 (25.0%)	15 (41.7%)	10 (27.8%)	9 (25.0%)	9 (25.0%)	205.56	0.03
*Taenia* spp.	0 (0%)	6 (16.7%)	0 (0%)	1 (2.8%)	0 (0%)	6 (16.7%)	0 (0%)	0 (0%)	0 (0%)	1 (2.8%)	377.81	0.0001
Hookworm	5 (13.9%)	7 (19.4%)	0 (0%)	5 (13.9%)	2 (5.6%)	9 (25.0%)	4 (11.1%)	6 (16.7%)	8 (22.2%)	2 (5.6%)	255.15	0.00005
*Ascaris lumbricoides*	0 (0%)	5 (13.9%)	0 (0%)	2 (5.6%)	0 (0%)	3 (8.3%)	0 (0%)	3 (8.3%)	0 (0%)	2 (5.6%)	327.26	0.049
Total positivity of wholesale markets					100 (46.30%)							
Total positivity of retail markets					131 (72.78%)							

## 4. Discussion

The present study revealed a high overall prevalence (56.3%) of intestinal parasitic contamination among vegetables collected from farmlands, wholesale markets, and retail markets in Hosanna and surrounding areas. This finding indicates that fresh vegetables represent a significant potential source of foodborne parasitic infections in the study area. The contamination rate observed in this study is higher than the pooled prevalence reported in Ethiopia (approximately 43%–44%) by recent systematic reviews and meta‐analyses, which documented substantial parasitic contamination of fruits and vegetables sold in local markets [4, 14]. The elevated prevalence may reflect poor sanitation conditions, use of contaminated irrigation water, and inadequate hygienic handling practices throughout the production and marketing chain.

The progressive increase in contamination from preharvest farms (35.42%) to wholesale markets (46.30%) and retail markets (79.44%) suggests that postharvest handling practices substantially contribute to vegetable contamination. Similar observations have been reported in Ethiopia, where contamination was associated with vegetable source, handling conditions, transportation, and market environments [3, 11]. The significantly higher contamination observed at retail markets may be attributed to prolonged storage periods, direct placement of vegetables on contaminated surfaces, lack of washing before display, and frequent contact by vendors and consumers. These findings underscore the importance of implementing hygienic practices throughout the vegetable distribution chain rather than focusing solely on farm‐level interventions.

Among the vegetables examined at the preharvest stage, Ethiopian kale exhibited the highest contamination rate (54.16%). The elevated contamination observed in Ethiopian kale may be attributed to its broad and uneven leaf surface, which favors the attachment and retention of parasite eggs, cysts, and larvae. The pattern of contamination observed in this study, where higher rates were recorded in leafy vegetables like Ethiopian kale as compared with smoother surface vegetables like carrots and beetroot, is in line with the findings of several other studies [8, 14]. The complex surface and large leaf area of kale and cabbage can facilitate the adherence of parasites. Among the parasites that were found, *E. histolytica/dispar* in Ethiopian kale at 33.3% and *S. stercoralis* in beetroot (33.3%) were the most prevalent. This was in agreement with a number of studies that stated *Entamoeba* spp. as the major protozoa, which contaminated about 6.4% of the vegetable samples [14]. Likewise, a survey in Arba Minch, southern Ethiopia, pointed out *E. histolytica/dispar* as the most common protozoan (8.4%) in the vegetable samples [11]. Moreover, Ethiopian studies have reported *Strongyloides* as one of the more frequently encountered helminths in vegetable contamination [14, 17].

The postharvest study on parasitic contamination examination of vegetables collected from the wholesale market revealed that carrot samples were heavily contaminated with parasites, 25 (69.44%), whereas tomato and cabbage had the least contamination, 6 (16.67%). Furthermore, a recent Ethiopian study reported similar contamination with 63.8% for carrots and 63.1% for lettuce, which are comparable to the 69.4% contamination of carrot samples in the present study [12]. On the contrary, tomato contamination in the current research (16.67%) was much less than the pooled estimate (~36%) and also lower than the cabbage contamination reported in other studies (~54%) [3]. The variation could be a result of disparities in local environmental conditions, hygiene practices in handling and transportation, water sources for irrigation, and detection methods used. The parasite *E. histolytica/dispar* was present in all types of vegetable samples, whereas *A. lumbricoides* was found only in Swiss chard samples. The differences in the prevalence of intestinal parasites between different studies could be due to various reasons, such as geographical variation, type of vegetables, vendors′ handling practices, transportation means from farms to markets, sample size, diagnostic methods, irrigation water quality, and postharvest conditions [3, 12].

The postharvest from retail markets in the present study exhibited several unhygienic practices: Vegetables were placed directly on the ground, there was a lack of washing before display, and protective equipment was absent among vendors. Out of the parasite‐positive retail market vegetable samples, *E. histolytica/dispar* was the most frequent contaminant (50.00%) detected from tomato and cabbage, followed by *S. stercoralis* (41.67%) from cabbage. The results of this study were in line with more recent findings, which indicated that *E. histolytica/dispar* was frequently present on market vegetables in Ethiopia and other countries, followed by helminths, among which *Strongyloides* and hookworm were also noted to be regular contaminants of leafy vegetables [3, 9, 11]. On the other hand, a different study revealed that the larvae of *Strongyloides* species (13.5%) were the most commonly found parasites in vegetables and the *E. histolytica/dispar* cysts (12.8%) were the second most prevalent [18].

The comparative analysis of parasite infestation of fresh vegetable samples from both wholesale and retail markets indicated that the postharvest stages were marked with a significant rise in contamination levels, with 46.3% of wholesale and 72.8% of retail market samples being positive. These findings suggest that contamination continues to accumulate during transportation, storage, and marketing. Similar observations have been reported in Ethiopia and Yemen, where vegetables sold in retail markets exhibited significantly higher contamination levels than those collected closer to production sites, with overall contamination rates ranging from 25% to 76.9% and the frequent occurrence of protozoan cysts and helminth eggs on fresh produce sold in local markets [7, 11, 19].

As for the distribution of parasite species, *E. histolytica/dispar* was the most commonly found protozoan in both wholesale and retail markets. This is in line with prior studies, which revealed a pooled prevalence of *E. histolytica/dispar* in vegetable/fruit samples of about 9.24%. The predominance of this protozoan is usually attributed to the employment of contaminated irrigation or wash water, lack of vendor hygiene, and absence of sanitation facilities in market areas. *S. stercoralis* was then the next most prevalent parasite, especially present in carrots and beetroots. This corresponds perfectly with the meta‐analysis, which found *S. stercoralis* to be the overall most common parasite (12.87%) in fresh vegetables [14]. The ubiquity of *S. stercoralis* in root and leafy vegetables might be a result of contamination from poor soil management and the capacity of its infectious larvae to persist in moist environments. This study has some limitations. Its cross‐sectional nature limits causal interpretation and seasonal trend analysis. Additionally, purposive sampling of farms and markets may affect representativeness. Microscopy‐based parasite identification may underestimate species‐specific prevalence due to morphological similarities among certain parasites. Future longitudinal studies incorporating molecular diagnostic methods are recommended.

## 5. Conclusion

The current study revealed that the fresh vegetables sold and grown in smallholder farms around Hosanna town were heavily infected with intestinal parasites. The findings demonstrated that contamination increased progressively along the farm‐to‐market chain, from preharvest farms to wholesale markets, and reached its highest level in retail markets. Contamination was more rampant in retail markets than in wholesale markets and small‐scale farms, suggesting that there is a greater risk of contamination with parasites during postharvest handling and marketing. *E. histolytica/dispar* and *S. stercoralis* were the most frequently detected parasites, although hookworm, *Taenia* spp., and *A. lumbricoides* were also identified. Significant variations in contamination levels were observed among vegetable types and market sources, with tomatoes, cabbage, Ethiopian kale, and carrots showing particularly high contamination rates. Statistical analysis confirmed significant associations between vegetable type and parasite occurrence. The study demonstrates that raw vegetables can serve as important sources of intestinal parasite transmission to consumers. Improving irrigation water quality, ensuring safe fertilizer use, strengthening hygiene and sanitation practices during production and marketing, and increasing awareness among farmers, vendors, and consumers are crucial for reducing contamination and safeguarding public health.

### 5.1. Recommendations

• Comprehensive health education must be provided to the farming community, market sellers, food handlers, and the general population to create awareness of the risk involved in the use of polluted water bodies for the irrigation of vegetables.
• Proper washing and disinfection of vegetables are highly recommended before consumption.
• Hosanna town administration should provide training for market sellers and food handlers on the proper washing, handling, and transportation of vegetables.
• Further study should be conducted on farm workers, market sellers, and food handlers to better establish the epidemiology of these parasites in Hosanna town.

## Author Contributions

**Gebremedhn Yohannes**, **Amha Gebremariam**, and **Yoseph Baraki** conceived and designed the study, conducted the experiments, analyzed and interpreted the data, and prepared the manuscript.

## Funding

No funding was received for this manuscript.

## Disclosure

All authors read and approved the manuscript.

## Ethics Statement

Ethical approval for this study was obtained from the Institutional Review Board (IRB) of Wachemo University. The ethical review committee comprised Dr. Alemayehu Getahun, Dr. Zelalem Petros, and Dr. Daniel Mekore. The study was approved under Ethics Approval No. WCU‐IRB/0099/24. Written/verbal informed consent was obtained from all participating farmers and market vendors prior to interviews. Participation was voluntary, and confidentiality was maintained throughout the study.

## Consent

The authors have nothing to report.

## Conflicts of Interest

The authors declare no conflicts of interest.

## Data Availability

All data and materials are stated in the manuscript.

## References

[1] Kareshk A. T. , Khodadai M. , Ramezani A. , Maraki F. , Zahrayi F. , and Nakhaei M. , Cross Sectional Survey of Parasitic Contamination in Vegetables Consumed in Birjand City in 2024, Modern Care Journal. (2025) 22, no. 1, 10.5812/mcj-157489.

[2] El Safadi D. , Osman M. , Hanna A. , Hajar I. , Kassem I. I. , Khalife S. , Dabboussi F. , and Hamze M. , Parasitic Contamination of Fresh Leafy Green Vegetables Sold in Northern Lebanon, Pathogens. (2023) 12, no. 8, 10.3390/pathogens12081014, 37623974. PMC10459145 37623974

[3] Zeynudin A. , Degefa T. , Belay T. , Mumicha J. B. , Husen A. , Yasin J. , Abamecha A. , and Wieser A. , Parasitic Contamination of Fresh Vegetables and Fruits Sold in Open-Air Markets in Peri-Urban Areas of Jimma City Oromia, Ethiopia: A Community-Based Cross-Sectional Study,, Plos one. (2024) 19, no. 3, e0290655, 10.1371/journal.pone.0290655, 38512863. 38512863 PMC10956833

[4] Girma A. , Aemiro A. , Alamnie G. , and Mulie Y. , Parasitic Contamination and Its Associated Factors in Fruits and Vegetables Collected From Ethiopia′s Local Markets: A Systematic Review and Meta-Analysis, Environmental Health Insights. (2024) 18, 11786302241307882, 10.1177/11786302241307882, 39703376. 39703376 PMC11656433

[5] Gurmassa B. K. , Gari S. R. , Solomon E. T. , Goodson M. L. , Walsh C. L. , Dessie B. K. , and Alemu B. M. , Contribution of Wastewater Irrigated Vegetables to the Prevalence of Soil-Transmitted Helminth Infection Among Female Farmers in Addis Ababa, Ethiopia, Tropical Medicine and Health. (2024) 52, no. 1, 10.1186/s41182-024-00604-5, 38845065. PMC11155033 38845065

[6] Laoraksawong P. , Bunkasem U. , and Pradidthaprecha A. , Prevalence of Intestinal Parasitic Contamination in Fresh Vegetables in Bangkok, Thailand, and surrounding areas: A cross-sectional survey, Parasite Epidemiology and Control. (2025) 29, e00416, 10.1016/j.parepi.2025.e00416. 39991528 PMC11847469

[7] Muqbel A. M. Q. and Binsaad A. J. A. , Parasitic Contamination of Vegetables in Selected Local Markets in Aden Governorate, Yemen, Electronic Journal of University of Aden for Basic and Applied Sciences. (2023) 4, no. 2, 187–198, 10.47372/ejua-ba.2023.2.251.

[8] Bilgiç F. , Öztürk E. A. , Babat S. Ö. , Babaoğlu A. , Erdoğan D. D. , and Korkmaz M. , Determination of Parasitic Contamination in Vegetables Collected From Local Markets in İzmir Province, Türkiye, Journal of Parasitology. (2023) 47, no. 2, 105–111, 10.4274/tpd.galenos.2023.51422. 37249114

[9] Tadege B. , Mekonnen Z. , Dana D. , Sharew B. , Dereje E. , Loha E. , Verweij J. J. , Casaert S. , Vlaminck J. , Ayana M. , and Levecke B. , Assessment of Environmental Contamination With Soil-Transmitted Helminths Life Stages at School compounds, households and open markets in Jimma Town, Ethiopia, PLoS Neglected Tropical Diseases. (2022) 16, no. 4, e0010307, 10.1371/journal.pntd.0010307, 35377880. 35377880 PMC9009776

[10] Yahaya O. , Bishop H. , Umar I. , Enoch A. , and Markus D. , Distribution Dynamics of Intestinal Parasites on Vegetables Sold in Egah Market Kogi State, Nigeria, Science World Journal. (2023) 18, 71–74.

[11] Alemu G. , Mama M. , Misker D. , and Haftu D. , Parasitic Contamination of Vegetables Marketed in Arba Minch town, Southern Ethiopia, BMC Infectious Diseases. (2019) 19, no. 1, 10.1186/s12879-019-4020-5, 31088390. PMC6515664 31088390

[12] Asfaw T. , Genetu D. , Shenkute D. , Shenkutie T. T. , Amare Y. E. , and Yitayew B. , Parasitic Contamination and Microbiological Quality of Commonly Consumed Fresh Vegetables Marketed in Debre Berhan Town, Ethiopia, Environmental Health Insights. (2023) 17, 11786302231154755, 10.1177/11786302231154755, 36798697. 36798697 PMC9926396

[13] Hajjami K. , Ennaji M. , Amdiouni H. , Fouad S. , and Cohen N. , Parasitic Contamination on Fresh Vegetable Consumed in Casablanca City (Morocco) and Risk for Consumer, International Journal on Science and Technology. (2013) 2, 543–549.

[14] Animaw Z. , Melese A. , Bedane D. , Tadesse B. , Degarege D. , Admasu F. , Jegnie M. , and Emmanuel B. , Prevalence, Pattern and Predictors of Clinically Important Parasites Contaminating Raw Vegetables and Fruits in Ethiopia: A Systematic Review and Meta-Analysis, BMC Infectious Diseases. (2024) 24, no. 1, 10.1186/s12879-024-10034-7, 39395940. PMC11470547 39395940

[15] Bekele F. , Shumbej T. , Dendir A. , Mesfin D. , Solomon A. , Jemal A. , and Alemayheu M. , Contamination Rate of Commonly Consumed Fresh Vegetables and Fruits With Parasites of Medically Importance in Wolkite and Butajira Towns of Gurage Zone, Southern Ethiopia, International Journal of Public Health Science. (2020) 9, no. 3, 211–215, 10.11591/ijphs.v9i3.20395.

[16] Su G. L. S. , Mariano C. M. R. , Matti N. S. A. , and Ramos G. B. , Assessing Parasitic Infestation of Vegetables in Selected Markets in Metro Manila, Philippines, Asian Pacific Journal of Tropical Disease. (2012) 2, no. 1, 51–54, 10.1016/S2222-1808(12)60012-7.

[17] Demisie K. N. and Melese D. M. , Assessment of Bacterial and Parasitic Contamination of Fruits Gathered From Specific Local Markets in Addis Ababa, Ethiopia, Frontiers in Sustainable Food Systems. (2024) 8, 1402898, 10.3389/fsufs.2024.1402898.

[18] Alemu G. , Nega M. , and Alemu M. , Parasitic Contamination of Fruits and Vegetables Collected From Local Markets of Bahir Dar City Northwest Ethiopia, 2020, Research and Reports in Tropical Medicine. 10.2147/RRTM.S244737 PMC7127876 32280295

[19] Zade S. , Upadhyay T. K. , Rab S. O. , Sharangi A. B. , Lakhanpal S. , Alabdallah N. M. , and Saeed M. , Mushroom-Derived Bioactive Compounds Pharmacological Properties and Cancer Targeting: A Holistic Assessment, Discover Oncology. (2025) 16, no. 1, 10.1007/s12672-025-02371-z, 40314874. PMC12048390 40314874

